# Relationship between brainstem neurodegeneration and clinical impairment in traumatic spinal cord injury

**DOI:** 10.1016/j.nicl.2017.05.026

**Published:** 2017-06-01

**Authors:** Patrick Grabher, Claudia Blaiotta, John Ashburner, Patrick Freund

**Affiliations:** a Spinal Cord Injury Center Balgrist, University Hospital Zurich, University of Zurich, Zurich, Switzerland; b Wellcome Trust Centre for Neuroimaging, UCL Institute of Neurology, University College London, London, UK; c Department of Neurophysics, Max Planck Institute for Human Cognitive and Brain Sciences, Leipzig, Germany; d Department of Brain Repair and Rehabilitation, UCL Institute of Neurology, University College London, London, UK

**Keywords:** Spinal cord injuries, Brain stem, Atrophy, Periaqueductal grey, Pyramidal tracts, Red nucleus, Quantitative MRI, Structural MRI

## Abstract

**Background:**

Brainstem networks are pivotal in sensory and motor function and in recovery following experimental spinal cord injury (SCI).

**Objective:**

To quantify neurodegeneration and its relation to clinical impairment in major brainstem pathways and nuclei in traumatic SCI.

**Methods:**

Quantitative MRI data of 30 chronic traumatic SCI patients (15 with tetraplegia and 15 with paraplegia) and 23 controls were acquired. Patients underwent a full neurological examination. We calculated quantitative myelin-sensitive (magnetisation transfer saturation (MT) and longitudinal relaxation rate (R1)) and iron-sensitive (effective transverse relaxation rate (R2*)) maps. We constructed brainstem tissue templates using a multivariate Gaussian mixture model and assessed volume loss, myelin reductions, and iron accumulation across the brainstem pathways (e.g. corticospinal tracts (CSTs) and medial lemniscus), and nuclei (e.g. red nucleus and periaqueductal grey (PAG)). The relationship between structural changes and clinical impairment were assessed using regression analysis.

**Results:**

Volume loss was detected in the CSTs and in the medial lemniscus. Myelin-sensitive MT and R1 were reduced in the PAG, the CSTs, the dorsal medulla and pons. No iron-sensitive changes in R2* were detected. Lower pinprick score related to more myelin reductions in the PAG, whereas lower functional independence was related to more myelin reductions in the vestibular and pontine nuclei.

**Conclusion:**

Neurodegeneration, indicated by volume loss and myelin reductions, is evident in major brainstem pathways and nuclei following traumatic SCI; the magnitude of these changes relating to clinical impairment. Thus, quantitative MRI protocols offer new targets, which may be used as neuroimaging biomarkers in treatment trials.

## Highlights

• Quantitative MRI revealed in-vivo brainstem neurodegeneration in SCI patients.
• Atrophy was evident in major sensorimotor brainstem pathways.
• The magnitude of myelin reduction in brainstem nuclei related to clinical disability

## 1 Introduction

Traumatic spinal cord injury (SCI) is a devastating condition and causes permanent sensorimotor loss and autonomic dysfunction in most patients, with no cure currently available (Dietz and Fouad, 2014). Usually patients show some degree of recovery which levels off within two years after injury. Using computational neuroimaging approaches, rapid and dynamic trajectories of neurodegenerative processes have been identified above the level of injury that accompanied the recovery. Crucially, the magnitude of neurodegeneration was associated with clinical impairment (Freund et al., 2013, Grabher et al., 2015).

Besides neurodegeneration at the spinal and cortical level (Beaud et al., 2008, Jirjis et al., 2015), retrograde and transneuronal degeneration has been shown in experimental SCI in brainstem pathways (Jirjis et al., 2015, Jones and Pons, 1998) and nuclei (Jones and Pons, 1998, Kwon et al., 2002, Wannier-Morino et al., 2008). The brainstem is phylogenetically highly conserved in mammals and plays a key role in motor (Lemon, 2008) and sensory function (Benarroch, 2012, Liao et al., 2015). Important substructures of the motor system entail the rubrospinal system (i.e. execution of precise limb movements), the vestibulospinal system (i.e. balance and posture), the reticular formation (i.e. initiates and coordinates limb movements and postural support), and the corticospinal system (i.e. skilled motor function) (Lemon, 2008), while the dorsal column nuclei and medial lemniscus (Liao et al., 2015) and the periaqueductal grey (PAG) (Benarroch, 2012) are involved in sensory processing and pain modulation. Crucially, structural reorganization of brainstem pathways and nuclei has been associated with functional recovery following experimental SCI (Zaaimi et al., 2012, Zörner et al., 2014). Thus, understanding trauma-induced pathophysiological processes affecting the brainstem pathways and nuclei might offer crucial insights into neurodegeneration and plasticity.

However, the brainstem is still understudied in human SCI as accurate and sensitive neuroimaging tools targeting the brainstem have only recently become available (Lambert et al., 2013b). First attempts using neuroimaging approaches provided evidence of brainstem atrophy (i.e. volume loss) (Freund et al., 2013, Freund et al., 2012, Grabher et al., 2015, Wrigley et al., 2009) and plasticity (i.e. volume increases) during intensive training (Villiger et al., 2015) in human SCI. Recent improvements in quantitative MRI (qMRI) techniques now allow quantification of the underlying microstructural changes (Weiskopf et al., 2015) and segmentation of individual brainstem pathways and nuclei (Lambert et al., 2013b). This is possible because different MR contrasts can be used to calculate quantitative maps (magnetisation transfer saturation (MT), longitudinal relaxation rate (R1), effective transverse relaxation rate (R2*)), which are sensitive to myelin (Schmierer et al., 2004, Turati et al., 2015) and iron (Stüber et al., 2014). Such maps can be used for multiparametric brainstem tissue segmentation (Lambert et al., 2013b). Myelin reductions have been shown to accompany atrophic changes in the cord and cortex, thus offering complementary insights into the sequela of SCI (Freund et al., 2013, Grabher et al., 2015). Furthermore, iron accumulation due to myelin breakdown has been reported in SCI (Kroner et al., 2014, Sauerbeck et al., 2013).

Here, we combined voxel-based quantification and multiparametric tissue segmentation to address our hypotheses that after traumatic chronic SCI, (1) atrophy and myelin reduction are evident in major brainstem pathways and nuclei and, (2) that the extent of atrophy, myelin reduction and iron accumulation relates to clinical impairment, lesion level and severity.

## 2 Methods

### 2.1 Participants and study design

We recruited 30 individuals with a chronic traumatic SCI (3 female) and 23 healthy participants (10 female) at the University Hospital Balgrist between August 2011 and May 2015. Fifteen patients were tetraplegic and fifteen paraplegic. All patients were treated surgically for decompression. No participant reported a history of medical, neurological, or psychiatric disorders and all were eligible for MRI examinations.

Patients underwent a comprehensive clinical protocol including (1) the International Standards for Neurological Classification of Spinal Cord Injury (ISNCSCI) (Kirshblum et al., 2011) to assess upper and lower extremity motor score (UEMS and LEMS), light touch (LT), pinprick (PP), lesion level, and severity (i.e. ASIA impairment scale (AIS)), and (2) the Spinal Cord Independence Measure (SCIM) (Catz et al., 2007).

To define the level of sensory and motor impairment, the most caudally intact dermatome for light touch and pinprick sensation (2/2 points) and motor function were considered, respectively (according to the ISNCSCI protocol). Lesion-level (neurological level of injury) was defined as the most caudal segment of the cord with intact sensation and motor function against gravity (min. 3/5 points), provided that motor and sensory function above this segment were normal. Lesion completeness was defined as having no motor and sensory function preserved in the sacral levels S4/5 (AIS A).

All participants gave informed written consent prior to study enrolment. The study protocol was in accordance with the Declaration of Helsinki and approved by the Ethics Committee of the Canton Zurich (reference number: EK-2010-0271).

### 2.2 Image acquisition

All participants' structural whole-brain data, including the cervical cord up to vertebra C5, were acquired on a 3T Magnetom MRI scanner (Siemens Healthcare, Erlangen, Germany). The system was equipped with a 16-channel radiofrequency (RF) receive head and neck coil and RF body transmit coil. A multiecho 3D FLASH (fast low-angle shot) sequence, with the following parameters, was used within a whole-brain multiparameter mapping (MPM) qMRI protocol (Draganski et al., 2011, Weiskopf et al., 2011): field of view (FoV) of 240 × 256 mm^2^, matrix size 240 × 256, isotropic resolution of 1 mm, GRAPPA parallel imaging in phase-encoding direction (anterior-posterior) with speed-up factor of 2, partial Fourier acquisition with 6/8 sampling factor in partition direction (left-right), and a readout bandwith of 480 Hz per pixel. Different weightings were predominantly achieved by choosing repetition time (TR) and flip angle (α): (1) T1-weighted (T1w): 25 ms/23°, (2) proton density-weighted (PDw): 25 ms/4°, and (3) MT-weighted (MTw): 37 ms/9° with off-resonance RF pulse prior to excitation. Echoes were acquired at seven equidistant echo times (TE) from 2.46 ms to 17.22 ms for all volumes, with an additional echo at 19.68 ms for PDw and T1w.

### 2.3 Image pre-processing

The acquired T1w, PDw, and MTw echoes were first averaged to increase the signal to noise ratio (SNR) and then used to calculate quantitative maps of MT and R1 (Draganski et al., 2011, Weiskopf et al., 2011) in MATLAB (MathWorks, Natick, MA). R2* was calculated from the log signal of the PDw echoes. UNICORT (Weiskopf et al., 2011) was used to correct RF transmit field inhomogeneity.

### 2.4 Brainstem template generation

We first generated brainstem tissue probability maps (TPMs) for the spatial alignment of brainstem sub-structures in our study cohort and to increase sensitivity for pathophysiological processes. Before generating the TPMs, we extracted the brainstem from quantitative maps from a longitudinal qMRI dataset of 29 subjects over four time points (Freund et al., 2013, Grabher et al., 2015) by label propagation using a set of brain labels (Neuromorphometrics Inc., Somerville, USA). Subsequently, whole-brain deformation fields were derived by segmenting the MT maps (Ashburner and Friston, 2005) and then applying a diffeomorphic image registration algorithm (Ashburner, 2007). The derived deformation fields enabled the extracted qMRI brainstem data to be transformed to the MNI space.

**Fig. 1:**
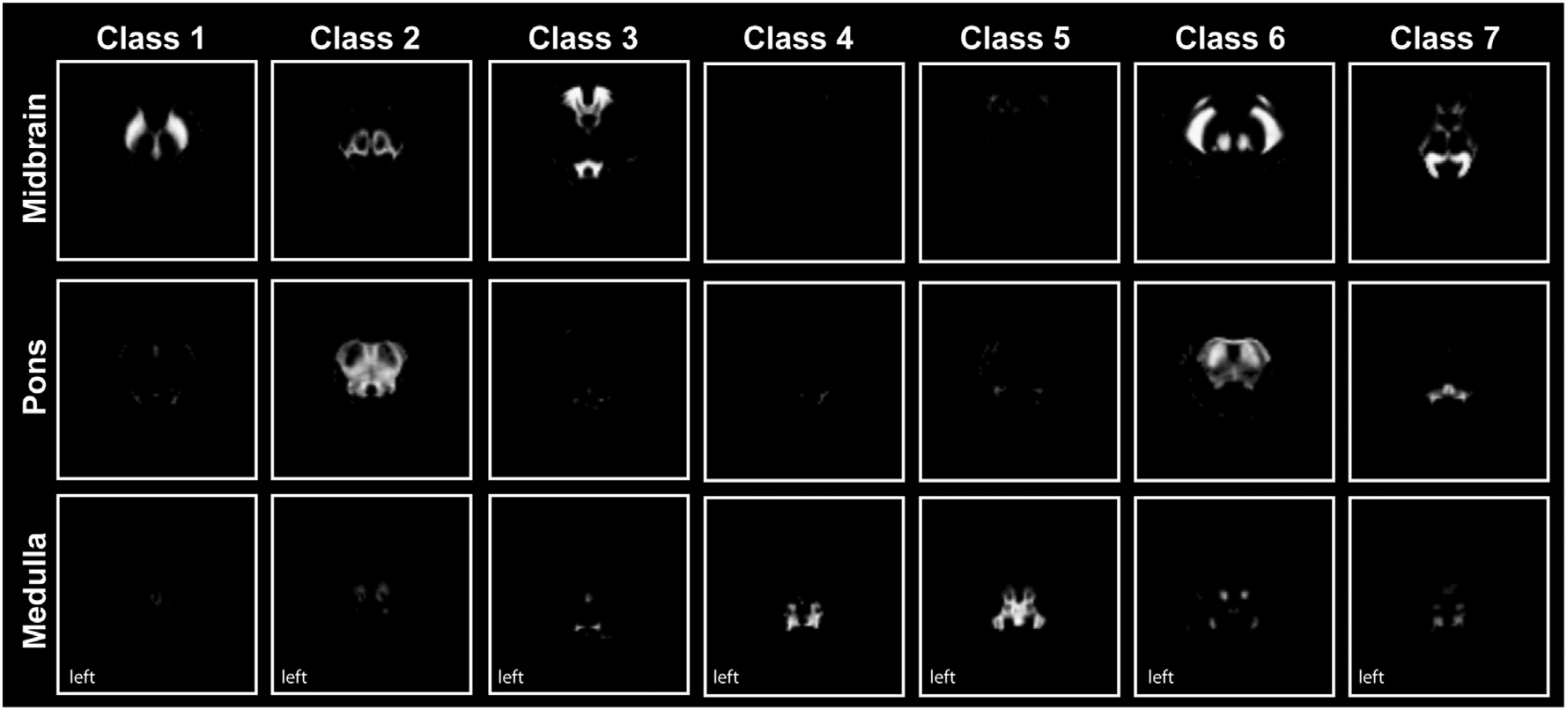
Brainstem tissue probability maps (classes 1–7). Seven within-brainstem tissue classes were derived from multiparametric brainstem segmentation using a multivariate mixture of Gaussians. They contain brainstem nuclei including the substantia nigra (class 1), the periaqueductal grey (class 3), and the red nucleus and cerebral crus (class 6).

We then used a multivariate Gaussian mixture model to generate brainstem TPMs (Hasselblad, 1966). Such a model assumes that the observed image intensities are drawn from a set of multivariate Gaussian probability density functions, where each Gaussian captures the intensity distribution of one single tissue type. Additionally, we introduced locally-varying, unknown tissue priors, which are learned directly from the observed data, thus providing a set of population-specific, average-shaped TPMs (Blaiotta et al., 2016, Lambert et al., 2013b). The statistical Gaussian mixture model was fit to the spatially normalized qMRI brainstem data, using the Expectation-Maximization algorithm (Moon, 1996), which is a general and well-established technique to obtain *maximum likelihood* or *maximum a posteriori* estimates of the model parameters, for probabilistic latent variable models. Within the neuroimaging community, such a method has been extensively validated for the classification of neural tissue types from MR data (Ashburner and Friston, 2005, Blaiotta et al., 2016, Lambert et al., 2013b). The resulting seven brainstem TPMs (classes 1–7) are shown in Fig. 1 and contained, amongst others, the red nucleus (RN) (class 6), cerebral crus including the corticospinal tracts (CSTs) (class 6), and PAG (class 3). Anatomical locations were validated using a high-field MRI brainstem atlas (Naidich et al., 2009). The tissue probability maps were subsequently aligned and merged with the whole-brain TPMs provided with SPM12 (http://www.fil.ion.ucl.ac.uk/spm/) (subsequently referred to as modified TPMs), so as to allow a more accurate alignment of the brainstem tissue maps with the individual scans, during the following processing steps. In fact, information derived from the tissues surrounding the brainstem (i.e. grey and white matter) can be effectively used to drive the registration of the individual volumes to the population mean, therefore ensuring more accurate segmentation results.

### 2.5 Voxel-based macrostructural and microstructural analysis of the brainstem

**Fig. 2:**
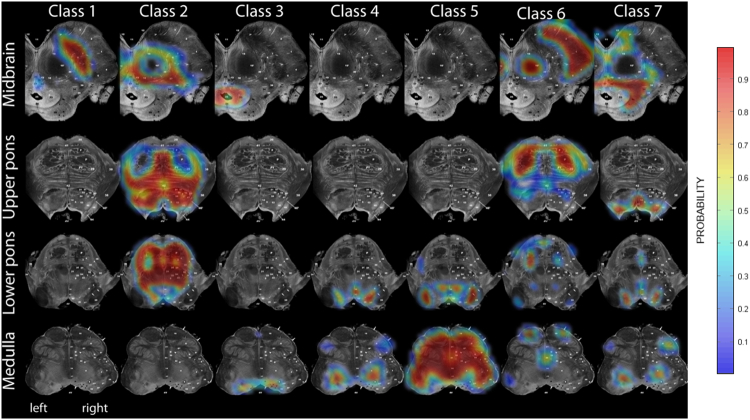
Overlay of study population mean onto high-resolution Duvernoy Atlas of the brainstem (Naidich et al., 2009). The population mean was derived by registration of the seven brainstem tissues of all subjects and are in correspondence to the obtained brainstem tissue probability maps (see Fig. 1).

We used our modified TPMs to segment the brain data (using MT and PDw data) of our study population (30 SCI and 23 controls) into grey matter, white matter, cerebrospinal fluid, plus the seven brainstem tissues for each subject (Ashburner and Friston, 2005). Then, a geodesic shooting registration algorithm (Ashburner and Friston, 2011) was used to align the seven brainstem tissues of all subjects and to create a common study population mean (Fig. 2). The estimated deformation fields were used both to compute Jacobian determinant maps for tensor-based morphometry (TBM), and to warp the quantitative maps of MT, R1, and R2* into the study population mean space for voxel-based quantification (VBQ) (Draganski et al., 2011). Due to the lack of gyrification of the brainstem and due to the highly accurate warping algorithm, no smoothing was applied to achieve higher spatial accuracy for small brainstem structures (Lambert et al., 2013a, Lambert et al., 2013b). Note that using unsmoothed data reduces sensitivity (i.e. fewer false positive results) and increases specificity due to Random Field theory being overly conservative at low smoothness (Nichols, 2012).

We used *t*-tests within the framework of the general linear model (GLM) to assess morphometric and microstructural differences between individuals with SCI and healthy controls, between tetraplegic and paraplegic patients, and between patients with complete and incomplete lesions. We used regression models to assess the relationship between morphometric and microstructural measures and neurological and functional impairment (AIS, lesion level, LEMS; UEMS, LT, PP, SCIM). Covariates of no interest included age, total intracranial volume and scanner upgrade to control for confounding linear effects in all GLMs (Barnes et al., 2010). Cluster-inference was performed using a cluster-defining threshold of p = 0.001 and a family-wise error (FWE) corrected threshold of p = 0.05 using Gaussian Random Field theory to account for multiple comparisons (Friston et al., 1994) within regions of interest (ROIs) derived from the seven brainstem TPMs. Only significant results (p < 0.05) corrected for FWE are reported. The ROIs (i.e. brainstem TPMs) were used to increase sensitivity for pathophysiological processes and specificity for anatomical locations in brainstem sub-structures (e.g. CST, RN, PAG).

## 3 Results

### 3.1 Patients' characteristics

The mean age of patients was 44.7 years (standard deviation (SD) 16.7, range 19.1–72.6) and their time since injury was 3.0 years (SD 5.4, range 0.7–23.8) (Table 1). Control subjects had a mean age of 36.9 years (SD 11.8, range 24.0–66.0) that was not statistically different when compared to patients (p = 0.052). Neurological and functional outcomes of patients were as follows: UEMS 42.4 (SD 11.3, range 14–50), LEMS 15.1 (SD 20.5, range 0–50), PP 58.0 (SD 28.1, range 13–112), LT 68.3 (SD 26.0, range 16–112), and SCIM 57.9 (SD 25.7, range 19–100).

**Table 1:** Clinical and behavioural data of 30 patients with chronic traumatic spinal cord injury at time of MRI data acquisition.

ID	Age (years)	Time since injury (months)	Completeness	AIS	Site of impairment (motor/sensory)	ISNCSCI LEMS	ISNCSCI UEMS	ISNCSCI Pinprick	ISNCSCI Light Touch	SCIM
1	28.97	12.07	Complete	A	C5/C4	0	14	13	16	19
2	43.19	15.73	Complete	A	C6/C4	0	25	18	20	37
3	21.01	12.33	Complete	A	C6/C5	0	23	26	53	34
4	31.69	10.27	Complete	A	C6/C5	0	26	20	33	30
5	19.08	13.5	Complete	A	C6/C7	0	23	33	33	37
6	33.59	12.2	Complete	A	C7/C7	0	35	29	32	26
7	60.36	68.17	Complete	A	T1/T1	0	49	40	52	32
8	52.76	54.6	Complete	A	T3/T3	0	50	44	47	53
9	26.13	10.8	Complete	A	T4/T4	0	50	46	48	67
10	70.28	9.5	Complete	A	T7/T7	0	50	68	67	49
11	39.24	9.33	Complete	A	T7/T7	0	50	58	60	65
12	53.12	8.03	Complete	A	T9/T9	0	50	66	68	69
13	30.58	10.27	Complete	A	T10/T10	16	50	78	82	80
14	36.44	185.47	Complete	A	T12/T12	4	50	78	78	70
15	54.65	18.63	Incomplete	D	C3/C3	49	42	94	62	84
16	48.13	12.13	Incomplete	D	C5/C3	47	35	97	98	98
17	68.91	285	Incomplete	D	T1/C3	40	49	78	69	NA
18	43.01	186.77	Incomplete	B	C6/C4	0	25	32	77	29
19	51.99	9.7	Incomplete	C	C7/C5	12	32	44	67	31
20	23.67	12.2	Incomplete	D	T1/C6	19	48	37	72	70
21	31.26	12.3	Incomplete	B	T1/C7	0	48	46	68	38
22	71.74	11.9	Incomplete	D	T1/T2	41	48	41	112	36
23	72.56	11.97	Incomplete	E	T3/T3^a^	50	50	112	112	97
24	31.29	12.33	Incomplete	B	T4/T4	0	50	46	74	54
25	28.9	22.83	Incomplete	B	T6/T6	0	50	52	77	66
26	53.11	11.97	Incomplete	D	T10/T10	48	50	90	90	100
27	68.84	12.17	Incomplete	B	T11/T11	32	49	74	92	42
28	32.49	10.77	Incomplete	B	T11/T11	0	50	72	78	66
29	44.82	13.4	Incomplete	D	L3/L4	45	50	106	106	100
30	68.21	12.07	Incomplete	D	S1/L3	50	50	102	107	100

AIS = ASIA impairment scale. ISNCSCI = International Standards for the Neurological Classification of Spinal Cord Injury. NA = not available.

a Initial level of injury as this patient has recovered (AIS E).

### 3.2 Volume loss and myelin reductions in brainstem pathways and nuclei

**Fig. 3:**
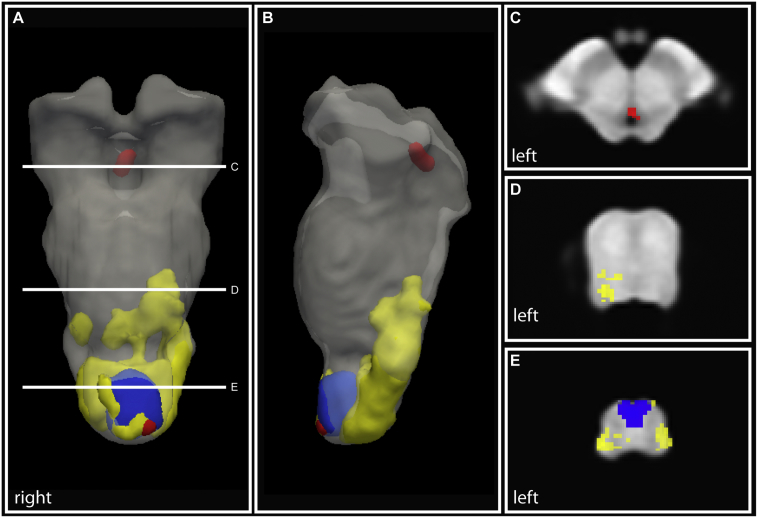
Volume loss and myelin reductions in brainstem pathways and nuclei in chronic SCI. (A & B) Three dimensional illustration of atrophy (Jacobians, blue) and myelin reductions (R1: yellow, MT: red) in the brainstem. Please note that for illustrative purposes the statistically significant clusters were smoothed with a Gaussian kernel with 1 mm full width at half maximum. Overlay of statistical parametric maps showing atrophy in the corticospinal tracts and medial lemniscus (E) and myelin reductions in the periaqueductal grey (C), in the dorsal pons (D), and in the dorsal medulla (E).

Voxel-wise analysis revealed significant atrophy and myelin reductions in patients compared to healthy controls within the brainstem (Fig. 3, Table 2). Volume loss was observed in the CSTs and medial lemniscus at the level of the medulla (p = 0.017). Lower myelin-sensitive MT was evident in the left CST at the level of the medulla (p = 0.039) and within the PAG (p = 0.001). Lower myelin-sensitive R1 was observed in the CSTs at the level of the medulla (cluster 1: p = 0.003; cluster 2: p = 0.025) and bilaterally in the dorsal medulla (p < 0.001), in the dorsal pontomedullary junction (cluster 1: p = 0.020; cluster 2: p = 0.038) and in the dorsal pons (7 clusters, Table 2). Iron-sensitive R2* did not reveal any significant changes in patients compared to controls. No between group differences were observed in volume and quantitative maps in tetraplegic and paraplegic patients and in patients with complete (i.e. AIS A) and incomplete lesions (i.e. AIS B-E).

**Table 2:** Group analysis showing volume loss and microstructural changes within the brainstem in individuals with SCI compared to healthy controls.

Modality	p-Value (FWE-corrected)	Cluster extent (voxels)	Z score	Coordinates (mm)			Anatomical location
				x	y	z	
Jacobian determinant (i.e. volume loss)	0.017	336	3.87	0	− 39	− 49	Cluster spanning the CSTs and medial lemniscus (medulla)
MT (i.e. myelin reduction)	0.001	15	4.32	1	− 28	− 4	PAG
	0.039	8	4.5	− 2	− 38	− 54	CST (L, medulla)
R1 (i.e. myelin reduction)	< 0.001	110	4.78	− 8	− 38	− 27	Low-mid pons (L, dorsolateral)
	0.019	21	3.77	− 8	− 40	− 29	Low-mid pons (L, dorsolateral)
	0.006	18	3.66	9	− 43	− 31	Low pons (R, dorsolateral)
	0.01	16	4.07	2	− 41	− 34	Low pons (dorsomedial)
	0.005	7	3.85	4	− 42	− 35	Low pons (R, dorsomedial)
	0.011	4	3.55	− 2	− 41	− 36	Low pons (L, dorsomedial)
	0.024	2	3.41	− 1	− 39	− 36	Low pons (L, dorsomedial)
	0.038	1	3.18	6	− 44	− 37	Pontomedullary junction (R, dorsolateral)
	0.02	13	3.82	2	− 43	− 39	Pontomedullary junction (R, dorsomedial)
	< 0.001	485	5.11	− 7	− 44	− 48	Medulla (bilateral, dorsal)
	0.025	12	4.99	4	− 35	− 49	CST (R, medulla)
	0.003	22	4.53	− 1	− 38	− 54	CSTs (bilateral, medulla)

CST = corticospinal tract, FWE = family-wise error, L = left, MT = magnetisation transfer saturation, PAG = periaqueductal grey, R = right, R1 = longitudinal relaxation rate.

### 3.3 Clinical impairment relates to neurodegeneration

**Fig. 4:**
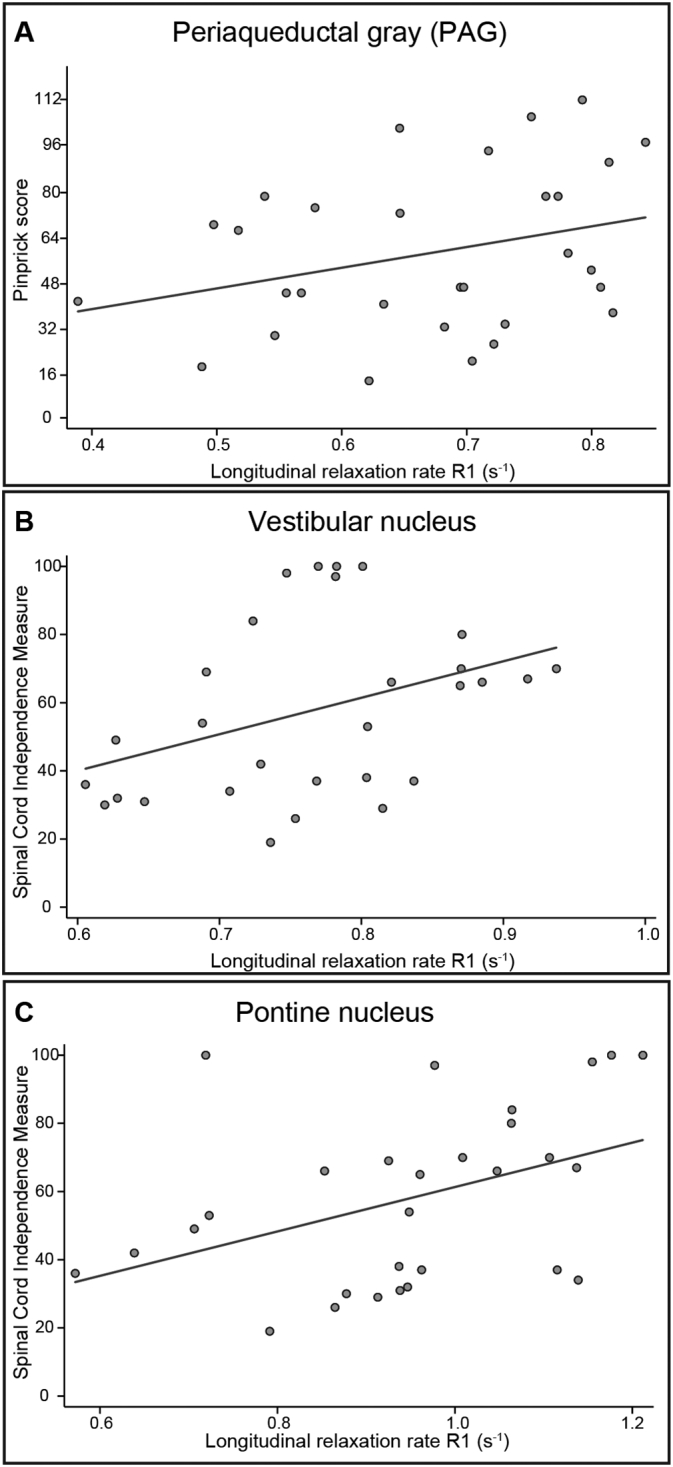
Correlation between microstructural integrity within the brainstem nuclei and neurological and functional impairment. Regression models from extracted peak-voxel within the significant cluster are shown for illustrative purposes only (not adjusted for age, scanner, and total intracranial volume).

Lower R1 in the PAG was associated with lower pinprick score in individuals with SCI (Fig. 4A, p = 0.015, cluster extent (k) = 13, Z score = 4.44, x = 0, y = − 38, z = − 7, n = 30). Lower R1 in the left upper dorsolateral medulla (i.e. vestibular nucleus) (Fig. 4B, p = 0.038, k = 1, Z score = 3.24, x = − 4, y = − 45, z = − 43, n = 29) and left upper-mid ventrolateral pons (i.e. pontine nucleus) (Fig. 4C, p = 0.034, k = 12, Z score = 3.88, x = − 13, y = − 22, z = − 23, n = 29) was associated with lower SCIM (i.e. functional independence).

## 4 Discussion

This study revealed atrophy and myelin reductions (i.e. MT and R1) within major brainstem pathways and nuclei involved in motor and sensory (dys-) function in chronic traumatic SCI. Interestingly, atrophy was observed only in the motor and sensory pathways, whereas myelin reductions occurred also in areas containing brainstem nuclei. Crucially, the magnitude of myelin reductions was related to the extent of motor and sensory impairment. Therefore, these structural alterations in the brainstem could be considered, if reproduced in longitudinal studies, as new targets to monitor impairment and complement assessments in clinical trials following SCI.

### 4.1 Motor system neurodegeneration

First, we confirmed atrophy, the endpoint of neurodegeneration, in the CST (Freund et al., 2013, Wrigley et al., 2009) and show quantitative myelin reductions in the same areas, which is suggestive of retrograde fibre degeneration (Beaud et al., 2008). Interestingly, we did not observe any macrostructural or microstructural changes in the rubrospinal system (i.e. RN). Phylogenetically, the formation of direct cortico-motoneuronal connections (i.e. CST) reaching as far as the lumbar enlargement (Lemon, 2008) has rendered the rubrospinal system less important for the control of movements in man, where fibres terminate in the high cervical cord (Nathan and Smith, 1982). Conversely after experimental SCI, the RN and the rubrospinal fibres, which reach more caudally into the spinal cord, show signs of neurodegeneration (Kwon et al., 2002, Wannier-Morino et al., 2008). Thus, these findings might illustrate the more dominant role of the CST in processes of neurodegeneration and plasticity in the context of functional recovery after human SCI (Dietz and Fouad, 2014).

We further observed myelin reductions in areas involved in motor control such as the cerebellar peduncles and ventromedial brainstem pathways (Lemon, 2008, Moulton et al., 2010), independent of atrophy. Reduced postural stability and increased risk of fall in incomplete SCI patients might result from extrapyramidal changes in the vestibular and reticular system (Liechti et al., 2008). Crucially, myelin reductions in the medulla (i.e. vestibular nucleus) and pons (i.e. pontine nucleus) were related to lower functional independence (i.e. SCIM score). Thus we provide evidence that the integrity of the pyramidal and extrapyramidal systems – crucial for postural control and movement coordination and hence functional independence – are disturbed in human SCI. Interestingly, no relationships between clinical impairments and atrophy were observed. Volume loss is rather unspecific to the underlying pathological processes and points to the sensitivity and accuracy of quantitative markers of the myeloarchitecture. Therefore, the extrapyramidal system, in combination with advanced neuroimaging tools, might offer new treatment targets, which could help to increase levels of independence in activities of daily living by improving postural stability (Liechti et al., 2008).

### 4.2 Sensory system neurodegeneration

Next to CST atrophy, we identified atrophic changes in the medial lemniscus. Although we did not observe any concomitant myelin changes, this is suggestive of anterograde fibre degeneration of sensory pathways (Jones and Pons, 1998). The PAG is part of the endogenous pain inhibition system and involved in motor function (Benarroch, 2012). We observed myelin reductions in the PAG which were directly related to impaired pinprick sensation (i.e. pain), a modality that conveys afferent information flow via the spinothalamic tract to second order neurons within the PAG. Altered information flow via the nociceptive dorsal horn neurons and the spinothalamic tract (Haefeli et al., 2013) into the PAG might therefore contribute to the development of neuropathic pain (Knerlich-Lukoschus et al., 2011), which is a condition that affects many SCI patients (Jutzeler et al., 2016). However, the clinical examination of pinprick sensation per se does not allow assessment of the directionality of this relationship. Therefore, it remains unclear whether the structural changes within the PAG are a result of primary loss of first order neurons or due to secondary processes affecting second order neurons, or both. Understanding the complex interaction of nociceptive (mal-) processing requires multimodal studies including direct readouts of nociceptive information flow (i.e. contact heat evoked potentials (Haefeli et al., 2013)) and structural as well as functional MRI. These multimodal biomarkers could then inform biophysical models of changes of states, which could be used to assess the directionality and extent of how the coupling between spinal cord, brainstem and brain is influenced by pain (Freund et al., 2016).

### 4.3 Iron content in brainstem pathways and nuclei

Interestingly, we did not observe iron accumulation caused by myelin breakdown (Hametner et al., 2013) causing detrimental inflammation in the CNS (Felix et al., 2012, Kroner et al., 2014). This may be due to rather small effects of iron accumulation in supraspinal regions compared to the spinal cord, where more iron is released by breakdown of haemoglobin after haemorrhage. Longitudinal assessment of iron accumulation after acute SCI will shed more light into these mechanisms with greater sensitivity to subtle effects.

### 4.4 Relationship between severity of injury and neurodegeneration

We did not observe either lesion level (tetra-/paraplegia) or severity (completeness) dependent neurodegeneration in brainstem sub-structures. This is in accordance with similar trajectories of atrophy above the level of injury between tetraplegic and paraplegic patients within the first year after injury within the spinal cord and brain (Freund et al., 2013). However, in chronic SCI lesion height and severity determined the magnitude of cord atrophy (Jutzeler et al., 2016). It therefore remains speculative why a cervical injury with a greater impact on the structural integrity of a higher number of fibres and neurons than a comparable thoracic lesion would not lead to more neurodegeneration within the brainstem. One potential explanation could be a flooring effect of atrophic progression due to the long-standing injuries. Moreover, given that the effect of trauma per se is strong, inducing linear and non-linear changes across the entire neuroaxis (Freund et al., 2013, Grabher et al., 2015), such changes, as well as differences in other factors (e.g. treatments, time spent in rehabilitation) and their complex interactions, may have concealed effects between patient sub-groups (i.e. tetra-/paraplegia, completeness). Finally, the cross-sectional design restricts conclusions to a single time point and fully characterizing these dynamic processes requires longitudinal studies. However, the results of the present study motivate the design of longitudinal studies with sophisticated neuroimaging protocols to establish these clinicopathological relationships.

Of note, quantitative measures of magnetisation transfer saturation and longitudinal relaxation rate provide information about macromolecular content within the neuronal tissue and are thus indirect measures sensitive to myelin. Post-mortem validation studies have shown the high association between MT-based measures and myelin as its main contributor (Schmierer et al., 2004, Turati et al., 2015). Although macromolecular content is the main component in R1, other contributors are water and iron content (Callaghan et al., 2015, Rooney et al., 2007). Finally, both quantitative readouts (MT and R1) are sensitive but not specific to a single underlying pathological mechanism. Thus they complement each other by providing insights into different disease processes (e.g. atrophy, change in myelin and water content, iron deposition) in brainstem sub-structures (Felix et al., 2012, Freund et al., 2013, Grabher et al., 2015, Kroner et al., 2014).

### 4.5 Limitations

We note the following limitations of this study. Patients were on average 7.8 years older than controls. Furthermore, age was significantly different between groups for pre-upgrade, but not for post-upgrade data. We therefore included age as a nuisance variable in all statistical models to account for linear age-related effects. The scanner was upgraded during the study period (from Verio to Skyra^fit^). Data from both patients and controls were acquired on both systems (the same ratio in each group) to minimize potential confounding effects due to the scanner upgrade. In addition, we used the scanner upgrade as nuisance variable in all GLMs. Due to the exploratory nature of this study, no adjustments for the number of contrasts tested were performed (Bender and Lange, 2001). The anatomical locations of brainstem ROIs (i.e. TPMs) and findings in SCI compared to healthy controls were carefully confirmed using a high-field MRI atlas (Naidich et al., 2009). The lack of specificity for pathological mechanisms influencing relaxation times and the small anatomical structures relative to the acquired resolution may conceal small effects and may be solved by high-resolution multiparametric MRI techniques (Weiskopf et al., 2015). The latter will help to improve segmentation of brainstem sub-structures and increase specificity for brainstem pathways and nuclei. The cross-sectional study design allows us to only assess differences in MRI readouts between SCI and healthy controls, but not the underlying trajectories of structural changes. This design is also less sensitive, as higher between-subject variability may conceal weak effects (e.g. no observed differences between tetraplegic and paraplegic patients, no dependence on lesion level and severity). Of note, lateralized findings are often introduced by thresholding statistical parametric maps to account for multiple comparisons. To overcome this limitation, we aim to develop a longitudinal analysis pipeline to assess trajectories of structural change in brainstem pathways and nuclei in acute SCI.

## 5 Conclusion

Refined qMRI methods enable tracking of spatially specific neurodegeneration and structural reorganization in the brainstem following traumatic SCI. Next to measures of atrophy, which are rather insensitive and unspecific to the underlying pathology, we show myelin reductions across the brainstem. Therefore, these clinically relevant structural brainstem alterations, obtained with a qMRI protocol, could serve as neuroimaging biomarkers to monitor treatment efficacy and complement clinical assessments in clinical trials following SCI.

## Funding

This study was funded by the Clinical Research Priority Program “NeuroRehab” of the University of Zurich and Wings for Life [WFL-CH-007/14]. The Wellcome Trust Centre for Neuroimaging is supported by core funding from the Wellcome Trust [091593/Z/10/Z]. Open access of this publication was supported by the Wellcome Trust.

## Conflicts of interest

We declare no conflicts of interest.

## Contributions

All authors were involved in the overall study design. PG analysed the data. CB and JA were involved in the methodological aspects of the study. PG and PF wrote the paper. All co-authors reviewed the paper.
